# Symmetric Reconstruction of Functional Liver Segments and Cross-Individual Correspondence of Hepatectomy

**DOI:** 10.3390/diagnostics11050852

**Published:** 2021-05-10

**Authors:** Doan Cong Le, Jirapa Chansangrat, Nattawut Keeratibharat, Paramate Horkaew

**Affiliations:** 1 School of Computer Engineering, Suranaree University of Technology, Suranaree, Nakhon Ratchasima 30000, Thailand; lcdoan@agu.edu.vn; 2 School of Radiology, Suranaree University of Technology, Suranaree, Nakhon Ratchasima 30000, Thailand; jirapa@sut.ac.th; 3 School of Surgery, Suranaree University of Technology, Suranaree, Nakhon Ratchasima 30000, Thailand; nattawut.k@sut.ac.th

**Keywords:** liver surgery, hepatectomy, preoperative simulation, computer-assisted intervention, conformal parameterization

## Abstract

Accurate localization and analyses of functional liver segments are crucial in devising various surgical procedures, including hepatectomy. To this end, they require the extraction of a liver from computed tomography, and then the identification of resection correspondence between individuals. The first part is usually impeded by inherent deficiencies, as present in medical images, and vast anatomical variations across subjects. While the model-based approach is found viable to tackle both issues, it is often undermined by an inadequate number of labeled samples, to capture all plausible variations. To address segmentation problems by balancing between accuracy, resource consumption, and data availability, this paper presents an efficient method for liver segmentation based on a graph-cut algorithm. One of its main novelties is the incorporation of a feature preserving a metric for boundary separation. Intuitive anatomical constraints are imposed to ensure valid extraction. The second part involves the symmetric conformal parameterization of the extracted liver surface onto a genus-0 domain. Provided with a few landmarks specified on two livers, we demonstrated that, by using a modified Beltrami differential, not only could they be non-rigidly registered, but also the hepatectomy on one liver could be envisioned on another. The merits of the proposed scheme were elucidated by both visual and numerical assessments on a standard MICCAI SLIVER07 dataset.

## 1. Introduction

Subject-specific modeling of 3D organs plays a crucial part in computerized diagnosis and therapeutic intervention. The process consists of digitizing a geometric representation of the organ from medical images, and performing its simulation in a clinically relevant setting [1]. Generally, the former involves the separation of objects of interest from the background, while the latter provides a platform where the user can interact with a virtual environment. In particular, simulation on virtual organs allows physicians to devise appropriate planning and to evaluate prognostic outcomes prior to the actual procedure. In living donor liver transplantation, for example, the accurate localization of the interested region and measurement of their future remnant volumes (FLRV) are among the determining factors of success in hepatic surgery. Thus far, the structure of a liver is not only complex but also greatly varies across individuals. Therefore, both 3D liver segmentation and its modeling remain important areas of investigation in biomedical research.

Despite rapid progress in artificial intelligence (AI) and image analysis, accurately determining specific anatomical structures from medical images remains challenging and is an open problem. This is primarily due to the fact that the task involves segmenting natural objects that greatly vary in both shape and size across individuals. In addition, the underlying imaging data are often of relatively low contrast. This results in a fuzzy edge and hence an indefinite object boundary. Furthermore, with certain imaging protocols and scanning planes, overlapping between objects could be present. Therefore, segmentation on a medical image remains highly dependent on the specific scenario and applications [2,3].

Recent advances in computer-assisted intervention (CAI) have so far enabled preoperative planning and simulation prior to an actual procedure, by modeling an organ of interest reconstructed from computed tomography (CT). Preoperative liver surgery, for example, has attracted much interest from the biomedical research community in recent decades. The liver is the biggest abdominal organ, and plays a crucial part in filtering and releasing toxic waste from the bloodstream. Consequently, it is particularly prone to various diseases such as hepatitis and cancer [3]. Therefore, in addition to surgical applications, segmenting a liver is considered a primal step toward many computer-aided procedures, e.g., diagnosis (CAD), chemical treatment, and other therapeutic interventions [4,5,6].

However, due to the highly complex anatomical arrangement in the abdominal area, a CT scan of the liver always includes other adjacent organs, such as the stomach, small and large intestines, pancreas, gallbladder, major vessels, and in some imaging studies, parts of cardiovascular structures. Due to such complexity, a liver may lie far from the focused region and alternately with other organs, whose tissue densities and thus pixel intensities are quite similar to those of the liver. Moreover, disease-induced morphological changes may as well alter the appearances of this already inhomogeneous structure [7,8,9,10,11]. It was widely acknowledged in the literature that these adverse factors contribute to poor accuracy and thus ineffective liver segmentation. While manually segmenting a liver by an experienced radiologist is tedious, time-consuming, and prone to observer variability, several computerized alternatives still rely, to some extent, on domain experts for the location, size, and general shape of a liver [9,12,13]. Nonetheless, an extensive investigation [14] on image segmentation has revealed great success in a range of modern clinical practices. While in general, those computerized methods offered accurate results and enabled much effective communication between physicians, others remain largely debatable whether an automatic or semi-automatic approach is the most suitable for a given problem.

Over the years, numerous techniques for segmenting a 3D liver from CT have been proposed. These techniques relied on different features being extracted from imaging data, and can be generally classified into those based on thresholding [11,15], region growing (RG) [13,16,17], graph processing [5,18,19,20,21,22], machine learning [23,24], level-set [25], and deformable model [26]. Detailed survey and discussion on the state of the art are presented in [27,28] and summarized as follows:

One straightforward approach was taken in [11], where thresholding was applied to an underlying image, and a liver was then estimated as the largest connected component (LCC). Subsequently, Bayesian classification was used to label individual pixels as either liver or non-liver a posteriori, based on their inferred type. Similarly, in [15] a single block linear detection algorithm (SBLDA) was proposed. The image was first preprocessed by median filter and morphological operator, whereby the block values and confidence matrix were respectively computed. Finally, the edges of the liver were extracted based on this matrix. Their major drawback was a high dependency on empirical thresholds, estimated from a limited dataset. To avoid such dependency, RG was chosen in some studies. Chen et al. [16] proposed liver segmentation from CT based on enhanced RG. Firstly, the image was preprocessed by a feature-preserving anisotropic filter. Then, a Gaussian mixture model (GMM) was utilized to estimate liver and non-liver statistics. Given seed points and their intensities as obtained from the previous slice, both regions were grown with respect to the GMM. Unfortunately, quantitative evaluation was not presented in that study. Kumar et al. [17] automatically specified a seed point as the centroid of binarized LCC, based on its grayscale histogram. Instead of relying on seed point priors, Lu et al. [13] introduced a quasi-Monte Carlo (QMC) method for seed point selection, thus improving the RG criteria. The segmented region was later post-processed by morphological operators, Canny edge detection, and the flood–fill algorithm. An alternative to thresholding and RG is the Graph Cut (GC) algorithm, introduced by Boykov et al. [29] for optimal boundary and region segmentation of an object via energy minimization. Later, the method was further enhanced by employing max-flow/min-cuts algorithms in minimization [30], and has since been widely received by many studies. Taking advantage of these state-of-the-art algorithms, Chen et al. [21] proposed combining the active appearance model, live wire, and GC for segmentation of the liver, kidneys and spleen. In liver analysis, Liao et al. [5] incorporated intensity and appearance models into the energy function of GC, to remove noise and highlight the region of interest (ROI). After that, the bottleneck detection algorithm was employed to refine the extracted liver boundary. Instead of bottleneck detection, another work [18] presented a vessel compensation method based on border marching in post-processing. Taking diseased samples into account, Peng et al. [19] considered a liver as multiple subregions, including those with a tumor. A proper region appearance constraint for each subregion was imposed onto the GC, in both boundary and region terms, and selected based on its geodesic distance. Their method, however, required the user initialization of cylindrical shapes, applied separately for healthy and diseased tissues. Alternatively, Wu et al. [20] proposed a super voxel-based GC method. After the usual preprocessing, maximum intensity projection (MIP) was used to estimate the volume of the abdominal region (VOA). Thresholding and morphological operators were then applied to extract the volume of interest (VOI). Finally, a simple linear interactive clustering algorithm was employed to construct, from this VOI, super voxels, on which the GC was optimized. Recently, Le et al. [22] highlighted a three-dimensional liver region based on multivariable normal distribution (MND). The resultant probabilistic map was enhanced by relaxation labeling before GC was employed to separate the liver from its background. In that work, the extracted contours were asserted by anatomical constraints. Deep machine learning has recently attracted considerable interest in the community. For instance, Lu et al. [23] adopted a three-dimensional convolution neural network (3D CNN) to build a liver probability map. Then, region appearance propagation (RAP) was used to model the intensity range of a liver. Based on this 3D CNN model, GC was finally used to segment the liver. Provided there was a sufficient manually delineated dataset, a deformable model such as level-set was found to be reliable and efficient [24,25]. Chartrand et al. [26] segmented a liver from CT and magnetic resonance imaging (MRI) based on this model, using a Laplacian mesh optimization scheme. With their method, an approximated 3D model of a liver was manually created via the graphical user interface (GUI). The model was then deformed toward an unseen liver instance by means of iterative non-rigid registration. An interactive correction, however, was needed for a user to interpret the results by imposing additional constraints on shape evolution.

The literature reviewed above may be categorized by the algorithm involved in their key steps. Nonetheless, a retrospective benchmarking of these methods is by no means trivial, due to large discrepancies among imaging modalities, datasets, empirical and environmental settings, and evaluation metrics. Table 1 summarizes the most recent liver segmentation methods, characterized by method, data involved, results, and automation.

Unlike MRI, imaging tissue with CT often exhibits ambiguous boundaries between adjacent organs, due to similar X-ray absorption. Priors on liver shape and size could very well offer a spatial cue in segmentation. Therefore, several studies exploited gradual variation across consecutive slices, covering the anatomical structure [5,16,18,20,26], to improve segmenting accuracy. For instance [5,18], localization from the previous slice was incorporated into the GC energy function to limit the search space. Meanwhile, spatial information within neighboring slices was exploited to seek suitable seed points [16], to determine VOI [20], or to build a 3D model from user-defined contours [26].

Despite several attempts to accelerate the process by automatic seed-point selection [17,18,20], if an underlying CT image includes multiple regions, its localization may be less accurate or even lie completely in non-liver areas (e.g., tumor, or dark object). It was, therefore, pointed out in [13] that interactive methods and those based on a statistical deformable model outperform their automatic counterparts, especially those without a prior model. Nonetheless, due to the particularly diverse morphology of a liver, a universal model would require a prohibitively large collection of training samples. On the other hand, with a limited number of known liver samples, higher interaction would be required on the user’s part. Depending on the specific purpose and expected degree of confidence, care must be observed when balancing these requirements and devising a liver segmentation scheme.

The remainder of this article is organized as follows. Section 2 presents the materials involved in this study and proposes a novel liver segmentation method. Section 3 describes in detail the experiments to evaluate this method. Then, Section 4 reports and discusses the findings. Finally, Section 5 offers concluding remarks and future works.

## 2. Materials and Methods

**Figure 1:**
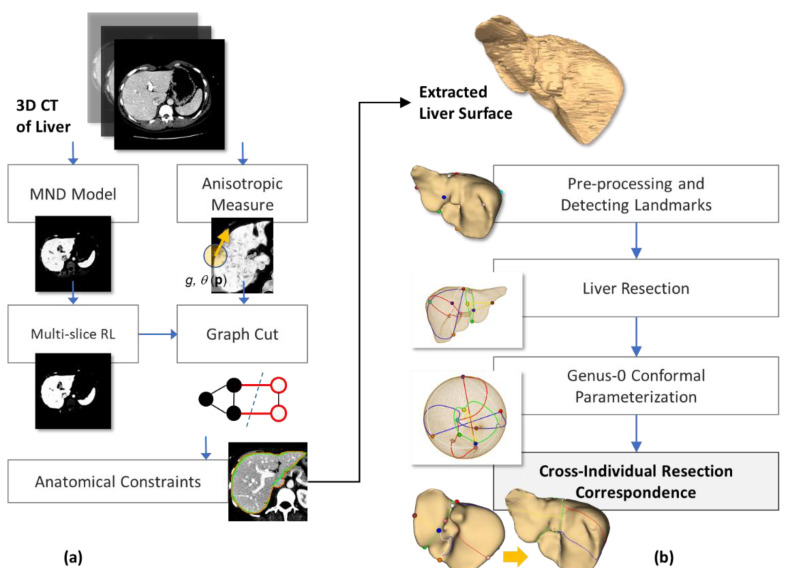
Diagram illustrating the overview of the proposed 3D liver segmentation method (**a**) and cross-individual modeling of hepatectomy (**b**).

This study proposes an efficient semi-automatic approach toward liver segmentation and its modeling from CT, consisting of the following steps. Firstly, local statistics (i.e., mean intensity, and standard deviation) within the neighborhood of each voxel were computed. Meanwhile, local orientation patterns (i.e., eigenvectors) and anisotropic metrics were also evaluated, at each voxel. A Gaussian mixture model (GMM) of tissue distribution was subsequently estimated from local pixel statistics. To minimize the inhomogeneity present in imaging data, and thus enhance multivariate probability estimation, iterative multi-slice relaxation labeling (MRL) was applied to this GMM. Later, both the enhanced probabilistic model and anisotropic metrices were incorporated into GC energy functions. Geometrical processing was then performed to remove any remaining anatomically unfaithful curve segments of the extracted contours, from which a 3D surface of a liver was finally reconstructed. Provided with the liver surfaces of different individuals, correspondence between their hepatectomy was established by using constrained conformal harmonic mapping. This process was summarized as a diagram in Figure 1.

In this paper, the extension and improvements of a previous work [22] are presented. Specifically, the MND model was built from multiple seed points (instead of one) to better capture the inhomogeneity of pixel intensities. Moreover, RL was extended to multiple slices, i.e., MRL (instead of within a slice), to improve the reliability of class assignments. Most importantly, a new feature-preserving metric was introduced and incorporated into the GC boundary term. Accordingly, the main contributions of this study are threefold. Firstly, the probability distribution of abdominal tissues, vital to most existing methods, was simply computed with no learning of features from other samples involved, but enhanced by contextual information, efficiently determined by MRL. Secondly, the local orientation pattern of underlying texture, instead of a generic Euclidean distance, was seamlessly incorporated into GC minimization, hence robustly reinforcing the morphological definition of a liver. Finally, and most importantly, the correspondence of hepatectomy between different subjects was determined by using conditioned conformal mapping of the extracted liver surfaces. This correspondence is potentially useful for functional cross-subject analyses, such as preoperative planning and the simulation of liver transplantation. Detailed analyses and treatments of the proposed method are described in the following subsections.

### 2.1. Probabilistic Model

Most existing studies exploited the probability distribution of an element based on extracted features. In [5,18] for example, an appearance model of a liver was built by means of principal component analysis (PCA), directly from pixel intensity. Instead of using this feature, other works derived a local binary pattern (LBP), a local variance of intensity (VAR) [19,23], or a GLCM [24] from an image pixel and its neighbors. With this approach, the extracted features were invariant to intensity level and local rotation. Similarly, our approach characterized each pixel with local statistical features, namely, means (*μ*) and standard deviations (*σ*) of multivariate Gaussian distribution. Given a seed point *p* placed inside a liver, a local patch *Ω_p_* centered at *p* was first located. Then, for each pixel *q* ∈ *Ω_p_*, local statistics, i.e., *μ_q_* and *σ_q_*, were computed within its local neighborhood, *Ω_q_*, and associated with *q*. Subsequently, a bivariate Gaussian model was created from these attributes around the seed point, *Ω_p_*, as given in Equation (1) [22]:

(1) $$\mathbb{P}\;\left(F_{q}\right)=\frac{1}{2\pi {\left|\Sigma \right|}^{1/2}}exp\left(-\frac{1}{2}{\left(F_{q}-\bar{F}\right)}^{T}\Sigma^{-1}\left(F_{q}-\bar{F}\right)\right)$$

where **F***_q_* is a feature vector at point *q*, i.e., $F_{q}={\left[\begin{array}{cc} \mu_{q} & \sigma_{q} \end{array}\right]}^{T}$**,** $\bar{F}$ is its average over a local patch *Ω_p_*, and Σ is a covariance matrix of these attributes. Thus, a total of (*N* + *N* − 1)^2^ pixels were involved in building the model for a seed point, where *N* is the width (or height) of *Ω_p,q_* [22]. As pointed out in earlier studies, the liver interior in a typical CT exhibits inhomogeneity, and hence results in an uneven probability map. To remedy this issue, we created separate distributions ${\mathbb{P}}_{i}\;\left(F\right)$ from *k* seed points, all within the liver. Then the probability of pixel *q* being associated with the liver is the maximum among these *k* distributions, i.e., ${\mathbb{P}}^{*}\;\left(F_{q}\right)=max\left({\mathbb{P}}_{0}\left(F_{q}\right),\ldots ,{\mathbb{P}}_{k-1}\left(F_{q}\right)\right)$. A background pixel would always have a low value, regardless. It is also worth noting that, while the inclusion of *Ω_q_* suppressed imaging noise (e.g., quantum mottle), the inclusion of *Ω_p_* tried to capture inhomogeneity, causing imaging artifacts and tissue variability. Increasing liver classes (*k*) could improve the robustness of the model, but at the cost of user interaction and optimal model selection. However, this model was used initially to normalize and highlight the liver appearance. This would be enhanced and classified in the subsequent modules. Thus, decisive intensity modeling was not particularly imperative at this stage. It was found from a preliminary experiment that *Ω_p,q_* of 9 × 9 pixels and *k* = 2 (i.e., a total of 2 × 17 × 17 = 578 pixels being evaluated) alleviated the abovementioned deficiencies and a balance between user interaction, the performance of model calculation and the reliability of the subsequent process, and was thus assumed in the experiments.

### 2.2. Relaxation Labeling (RL)

As its name suggests, relaxation labeling (RL) iteratively assigns class labels to object elements based on their contextual information [31]. Provided with a set of initial membership estimates, its probabilities of belonging to specified classes are gradually adapted to the surrounding compatibilities and supports, until convergence. Considering both spatial and probabilistic contexts, RL is hence among the most robust post-processing methods for various classification problems. To name a few, it was integrated into enhancing line and curve estimation [32], edge detection [33], image segmentation [34,35], point matching [36], and many others [37,38]. The formulation of an RL framework is described as follows.

Let an object element *p* ∈ 𝒫 be labeled, at a given iteration, *t*, to a class a∈C, with a probability, $Pr_{p}^{t}\left(a\right)\in \left[0,1\right]$, where $\underset{a\in C}{\sum }Pr_{p}^{t}\left(a\right)=1$. This probability will be updated at the next iteration, *t* + 1, following the expression in Equation (2) [31]:

(2) $$Pr_{p}^{t+1}\left(a\right)=\frac{Pr_{p}^{t}\left(a\right)\left(1+S_{p}\left(a\right)\right)}{\sum_{b\in C}Pr_{p}^{t}\left(b\right)\left(1+S_{p}\left(b\right)\right)}$$

where $S_{p}\left(a\right)$ is the support for an object *p* being assigned to a class, *a*. It was in turn expressed as a weighted sum among the compatibilities between object element *p* and its neighbors, *q*, being assigned to classes a and b, respectively, that is:

(3) $$S_{p}\left(a\right)=\underset{q\in N_{p}}{\sum }w_{pq}\underset{b\in C}{\sum }r_{pq}\left(a,b\right)Pr_{q}^{t}\left(b\right)$$

where the weight, *w_pq_*, satisfying $\underset{q\in N_{p}}{\sum }w_{pq}=1$, is the contribution of *q* on *p*, and $r_{pq}\left(a,b\right)$ is a compatibility between *p* and *q* (neighbor of *p)* being assigned to classes *a* and *b*, respectively. It returns 1 (fully compatible) if both *p* and *q* were assigned to the same class (i.e., a=b), and −1 otherwise (incompatible). Note that the denominator in (2) ensures the unity sum of all probabilities. A detailed discussion of the accuracy and convergence properties of these values can be found in [37].

### 2.3. Multislice Relaxation Labeling (MRL)

Motivated by previous works [5,16,18,20,26], where 3D information gathered from consecutive slices was exploited to remedy the ambiguity found in an individual CT image, this study took a multi-slice approach to RL (MRL). Since the liver is the largest abdominal organ, a gradual change of its morphology across adjacent slices may be incorporated into labeling contexts to improve the effectiveness of RL. To this end, it is simply expressed as an inverse Euclidean distance between voxel *p* and *q*, as in Equation (4):

(4) $$w_{pq}={\left[{\left(x_{p}-x_{q}\right)}^{2}+{\left(y_{p}-y_{q}\right)}^{2}+D^{2}{\left(s_{p}-s_{q}\right)}^{2}\right]}^{-1/2}$$

where *s* and *D* are the slice index and the distance between consecutive slices, respectively.

In this study, 26-neighbor (9 + 8 + 9) connectivity was considered for each voxel, *p*. The slice distance, *D*, can be found in DICOM metadata. It can be noted in Equation (4) that as objects become further apart, the weight gets weaker and hence there are fewer supports between them. In addition, as emphasized in [31], the weight $w_{pq}$, must be normalized over all neighbors so that it satisfies $\underset{q\in N_{p}}{\sum }w_{pq}=1$. To further speed up the calculation, all possible weights for 26 neighbors were precomputed and stored in a look-up table (LUT). The implementation of MRL was similar to [31], except for the calculation of multi-slice weight.

**Figure 2:**
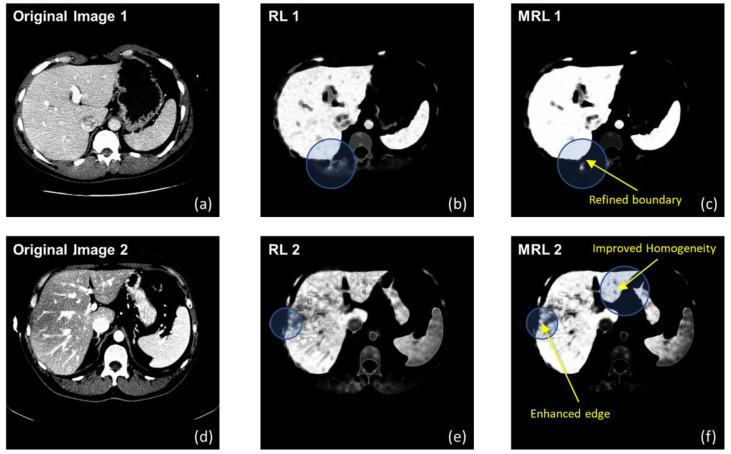
Labeling examples of two selected slices (**a**,**d**), given by a non-RL (**b**,**e**) and MRL (**c**,**f**). The circles highlight areas of improvement, which are of a refined boundary and much more homogeneous regions.

Figure 2 illustrates some soft labeling examples, comparing between results obtained by a non-RL, and homogeneity improvement made by MRL.

It is evident that the liver boundaries are refined, while artifacts are reduced. Unlike other similar works, MRL was used in this study only as preprocessing, and thus definitive binary classification was not fully committed as yet. Therefore, instead of continuing the updates until convergence, these examples were obtained after only five iterations.

### 2.4. Local Orientation and Anisotropic Measure

A CT image has unique textural inhomogeneity, which contributes to artifacts in segmentation. Several methods commonly resolve this issue by applying a low-pass filter or morphological operator, but inevitably smoothing out parts of the anatomical feature. Advanced feature-preserving filters were also proposed to remedy this shortcoming. Yang et al. [39], for example, proposed structural adaptive anisotropic filters to remove these artifacts, without much scarifying of image fidelity. With this technique, a convolution kernel was created specifically for an individual pixel, so that its orientation and shape were adaptive to those of the underlying pattern. Given an image *f*: **R**^2^ → **R**, the kernel at a pixel *p*, located at (*x*, *y*), is oriented along the local intensity pattern at an angle, *θ*, as given in Equation (5):

(5) $$\theta \left(p\right)=\frac{1}{2}atan\left(\frac{\iint_{N_{p}}^{}2f_{x}f_{y}dxdy}{\iint_{N_{p}}^{}\left(f_{x}^{2}-f_{y}^{2}\right)dxdy}\right)+\frac{\pi }{2}$$

where *f_x_* and *f_y_* are the 1st order partial derivatives of the image, *f*, with respect to *x* and *y* coordinates, respectively. The local neighborhood of *p* is denoted as $N_{p}$.

Then, the anisotropic measure and corner strength, used to estimate the kernel shape is given for each pixel, *p*, respectively, as follows:

(6) $$g\left(p\right)=\frac{{\left(\iint_{N_{p}}^{}\left(f_{x}^{2}-f_{y}^{2}\right)dxdy\right)}^{2}+{\left(\iint_{N_{p}}^{}2f_{x}f_{y}dxdy\right)}^{2}}{{\left(\iint_{N_{p}}^{}\left(f_{x}^{2}+f_{y}^{2}\right)dxdy\right)}^{2}}$$

(7) $$c\left(p\right)=\left(1-g\left(p\right)\right){\left|\nabla f\left(p\right)\right|}^{2}$$

where the partial derivatives are defined as in Equation (5) and ∇f(p) is an image gradient at pixel *p*. Finally, the shape kernel is defined by an ellipse, whose major and minor axes are denoted by *s*_1_ and *s*_2_, respectively, as expressed in Equations (8a) and (8b):

(8a) $$s_{1}\left(p\right)=\frac{r}{1+c\left(p\right)/\lambda }$$

(8b) $$s_{2}\left(p\right)=\left(1-g\left(p\right)\right)s_{1}\left(p\right)$$

where *r* is the support radius of a kernel, which is set to three in this paper, and *λ* is a factor controlling the importance of the imaging feature. The latter is normally set to 75% (0.75). For an isotropic region, *s*_1_ = *s*_2_ and *g* ≈ 0, then the kernel shape is circular, whereas for a structured region, *s*_1_ > *s*_2_ and 0 < *g* ≤ 1, then the kernel is elliptical.

It is worth emphasizing that, unlike in most previous works, the anisotropic metrices derived here were not intended to construct a smoothing or noise removal filter but were incorporated into GC energy functions for structural adaptive segmentation. Their detailed treatment and analysis are given in the following subsection.

### 2.5. Graph Cut Algorithm

Graph Cut (GC) poses the segmentation problem as graph optimization. Consider an undirected graph G={V,ℰ}, where V is a set of vertices and ℰ is that of edges connecting pairs of vertices, each by a positive weight, w. A minimum-cut (or min-cuts) scheme partitions  V into 2 disjoint subsets, i.e., $V_{1}$ and $V_{2}$ ($V_{1}\cup^{}V_{2}=V\;\wedge \;V_{1}\cap^{}V_{2}=\emptyset$), such that a cost function $\left|\;K\right|=\underset{e\subset K}{\sum }w_{e}$, defined on edges, is minimized, where K⊂ ℰ.

In image segmentation, V and  ℰ correspond to pixels (𝒫) and their adjacent links, respectively. An additional two special terminals, representing object and background are added to this graph, and thus are referred to as *s* (source) and *t* (sink), respectively. A link in this graph is categorized into either an *n*-link or *t*-link, whether it connects adjacent pixels or these pixels with terminals, respectively. Let *p* be a pixel within an image 𝒫, whose size is ||𝒫||. A pixel *p* is assigned with a binary label *l*_p_, i.e., $l_{p}\in \;ℒ=\left\{0,1\right\}$, where 0 and 1 denote the object and background classes, respectively. An efficient min-cut/max-flow [30] solves this min-cut problem by minimizing the combinational energy, given optimal labels, i.e.,

(9) $$E\left({ℒ}^{*}\right)=\alpha \underset{p\in 𝒫}{\sum }R\left(l_{p}\right)+\left(1-\alpha \right)\underset{p\in 𝒫,\;q\in N_{}\;}{\sum }B\left(p,q\right)\times T\left(l_{p},l_{q}\right)$$

where *α* is an empirical weight balancing between region (*R*) and boundary (*B*) terms, empirically set to 0.6 in the experiment. The greater the value, the more emphasis is placed on the former. The former is defined by the likelihood of specific labeling, while the latter is computed from the functional (i.e., intensity) and spatial differences between two adjacent pixels, i.e., the function *T* returns 1 if $l_{p}\neq l_{q}$, or 0 otherwise. One of the main contributions of this study is introducing local pattern compatibility to enhance the differences in the boundary. It is characterized by an underlying structural adaptive measure, *F*, between two pixels. Accordingly, the region and enhanced boundary terms of GC energy are given in Equations (10a) and (10b), respectively:

(10a) $$R\left(l_{p}\right)=1-Pr\;\left(l_{p}\;|\;p\right)$$

(10b) $$B\left(p,q\right)=exp\left(-F\left(p,q\right)\times \frac{{\left|I\left(p\right)-I\left(q\right)\right|}^{2}}{2\delta^{2}}\right)\times \frac{1}{||p-q||}$$

where *I* ( ∙ ) is the image intensity function, *δ* is the noise standard distribution, and ||∙|| is the Euclidean norm in a Cartesian space. The symmetric structural adaptive measure of pixels *p* and *q* is the maximum anisotropic function (Equation (6)) between the two, that is,

(11) F(p,q)=max(g(p),g(q))

### 2.6. Post Processing of Extracted Contours

Segmenting a liver based on pixel-wise information is robust against imaging noise. Thus far, it does not take into account the anatomical contexts. To address the issue, this study incorporated prior knowledge on liver shape into post-processing. A set of parallel contours were first extracted from the probability maps, by using Otsu’s method, and had its contours extracted. Provided with these contours, two relevant constraints were imposed, i.e., a gradual transition between adjacent slices and, most importantly, a singly connected curve with no excessive nodule. The latter was usually contravened by a fuzzy boundary adjoining other abdominal organs. Their detailed analyses and treatments are described in the subsections below.

#### 2.6.1. Bottleneck Detection

A small nodule folded on a contour is characterized as a bottleneck (BN) [40] and on the 2D space can be identified by a pair of points (*p_i_*, *p_j_*) on a contour *C* for *i* ≠ *j*, that satisfies:

(12) $$\epsilon =\frac{||p_{i}-p_{j}||}{MIN\left(\oint_{p_{i}}^{p_{j}}C\left(s\right)ds,\oint_{p_{j}}^{p_{i}}C\left(s\right)ds\right)}<T_{b}$$

where *ϵ* is the bottleneck ratio, ||∙|| is the Euclidean norm, contour integrals are evaluated both clockwise and counterclockwise, and *T_b_* is a threshold. In addition, to prevent mistaking fissures for ligamentum teres and venosum for BN, only pairs (*p_i_*, *p_j_*) whose distances were greater than 60 pixels were considered.

It differed from [5] that, we adopted an outer angle criterion (*T_c_*) to limit the search space [22]. A point *p* whose outer angle was less than *T_c_* would be discarded from potential BN, and hence not detected by Equation (12). In the following experiments, *T_b_* and *T_c_* were empirically set to 0.6 and 135°, respectively. Moreover, a contour was first decimated by polygonal approximation, prior to being detected for BN to reduce processing time.

#### 2.6.2. Interslice Contour Constraint

The previous BN was intended for small knot removal, based on in-plane angles and distances. A slight deviation from empirical thresholds could lead to inconsistent removal between slices. Thus, to further improve the robustness of contour post-processing, we introduced interslice constraint. It imposed the minimum overlap ratio between two consecutive contours, to ensure natural transition along the liver cross-sections.

Starting with a slice *k*, containing contours with the largest total areas, *S* ($C_{k})=\underset{i}{\sum }S\left(c_{ki}\right)$, where *c_ki_* is the *i*th contour in that slice and *S* ( ∙ ) is an area function. While traversing along the *z*-axis in ascending order, let $\;C_{i}=\left\{c_{i1},c_{i2},\ldots \right\}$ and $C_{j}=\left\{c_{j1},c_{j2},\ldots \right\}$ be the set of contours (with BN removed) in current (*i*) and previous (*j*) slices, respectively. A contour, *c_is_* in $C_{i}$, was maintained only if there existed *c_jt_* in $C_{j}$ that the ratio between the area of their intersection, $S\left(c_{is}\cap^{}c_{jt}\right)$, and the minimum area between them, $min\left(S\left(c_{is}\right),S\left(c_{jt}\right)\right)$, were greater than a specified threshold, *T_s_*. Following [22], the threshold, *T_s_*, was set to 0.8–0.05 (D − 1), where *D* was the slice distance, as defined in Section 2.3. The process was also repeated, while also traversing in descending order along the *z*-axis.

### 2.7. Conformal Parameterization of a Liver Surface

The parameterization of a discrete surface defines a one-by-one mapping of 3D triangular mesh onto a topological homeomorphism, such as a plane or a unit sphere. This mapping must hold two characteristics, i.e., minimized distortion and (relative) area preservation [41]. Mesh parameterization has been used in a range of applications in image analyses and computerized graphics, e.g., texture mapping, occlusion completion, mesh compression, and shape metamorphosis [42]. In medical imaging, it is useful in analyzing and comparing biological materials, such as the brain, carotid artery and hippocampus [43], and the modeling of muscles, such as those of the levator ani [44] and face [45,46]. It has been commonly accepted that conformal mapping minimizes the angular distortion of surface elements, and thus their geometrical formation [47,48]. It has been demonstrated elsewhere [42,47,48] that, when provided with a mesh with specified topology, it can be mapped onto a structure with the same diffeomorphism, regardless of its geometry. However, particularly for anatomical shapes, a conformal mapping between two genus-0 surfaces is not unique but forms a Möbius group. Moreover, the alignment of their salient features is not necessarily guaranteed. To resolve this uncertainty, optimized Möbius transformation by explicit landmark matching or by modified mesh energy function was proposed [49,50]. While they both created conformal mapping with correspondences, the latter is superior in terms of alignment error, and was hence adopted in this study.

To define an optimal mapping between two liver surfaces *S*_1_ and *S*_2_, let *K* denote a triangular simplex of a liver surface and [*u*, *v*] be an edge connecting two vertices, *u* and *v*. Then, let $f_{s}:\;S_{s}\to S$ be the conformal map of a surface $S_{s,\left(s=1,2\right)}$ onto a unit sphere; $p_{i,i=1\ldots n}$, and $q_{i,i=1\ldots n}$ are landmarks defined on both surfaces, respectively, where *p_i_* corresponds to *q_i_*. Following [49,50], the harmonic energy function that minimizes both distortion and landmark differences is given by Equation (13):

(13) $$E\left(f_{1}\right)=\underset{\left[u,v\right]\in K}{\sum }k_{uv}||f_{1}\left(u\right)-f_{1}{\left(v\right)||}^{2}+\gamma \underset{1\leq i\leq n}{\sum }||f_{1}\left(p_{i}\right)-f_{2}{\left(q_{i}\right)||}^{2}$$

where $k_{uv}=\cot\alpha +\cot\beta$ with α and β are the opposite angles of an edge [*u*, *v*]; γ is a balancing factor. The correspondence, which registered *S*_1_ to *S*_2_, is given by $g=f_{2}^{-1}\circ f_{1}$. It may be observed that Equation (13) can be scaled to different mesh sizes, ||K||, and does not depend on physical coordinates. Therefore, this mapping could handle variability in both the shape and size of the liver.

It was pointed out [51] that with conventional approaches, numerical instability may occur near the spherical poles (where a large number of vertices are mapped onto a small region), and near the landmarks. These result in severe distortion and even loss of bijection. To resolve these issues, a Gauss map [48] was first applied to initialize uniform distribution, then the Fast Landmark Aligned Spherical Harmonics (FLASH) proposed by Choi et al. [51] were utilized. This linearized Equation (13), based on the north-south poles iterative model, while adjusting the Beltrami differential to recover bijective mapping. The detailed implementation of FLASH can be found in [51].

In this study, a liver surface was assumed topologically spherical and hence, of genus-0 form. Thus, it was possible to determine a conditioned conformal map, when provided with a few anatomical landmarks, that aligned two corresponding liver surfaces (e.g., that of donor and recipient). The potential application of such low-distortion mapping is demonstrated in a simulation by faithfully projecting functional resections of one liver onto another during a preoperative hepatectomy.

## 3. Experiments

### 3.1. Liver Dataset

To elucidate its merits, the proposed scheme was validated on the standard MICCAI-SLIVER07 dataset. This dataset comprised 20 CT scans of 3D livers with ground truth [12]. All scans were acquired by different scanners, but at the same 512 × 512 pixel resolution. Depending on volumes of interest, the number of slices and their distances (*D*) varied from 64 to 502 and from 1.0 to 3.0 mm, respectively. In-plane resolution (i.e., pixel spacing) varied from 0.55 × 0.55 mm^2^ to 0.8 × 0.8 mm^2^.

### 3.2. Evaluation Metrics

To benchmark against state-of-the-art methods, the extracted liver was evaluated by means of five standard metrices, i.e., volumetric overlap error (VOE) [%], relative volume difference (RVD) [%], average symmetric surface distance (ASD) [mm], root means square symmetric surface distance (RMSD) [mm], and maximum symmetric surface distance (MSD) [mm]. VOE and RVD measured the volumes of intersection and of difference between the reference and segmented livers. The latter could have negative or positive values, depending on whether a liver was under- or over-segmented. ASD, RMSD, and MSD measured the average, root means square, and maximum distances between a point on the reference liver to the segmented one and vice versa. For all the metrics, the lower the value, the higher the performance. It should be noted that zero ASD does not necessarily mean that both reference and segmented liver had 100% overlap, but it should be used in combination with other metrics. For comparison purposes, each error metric was converted to a score of 100 by using Equation (13), where ε and $\bar{\varepsilon }$ are the error and its average of a given metric, respectively [12]:

(14) $$\emptyset =\max\left(100-25\frac{\varepsilon }{\bar{\varepsilon }},0\right)$$

### 3.3. Implementation and System Environment

The proposed method was written in C/C++ languages and executed on an Ubuntu Linux system, equipped with an Intel Core i7 CPU and 16 GB RAM. Basic image processing operations, e.g., Otsu’s method, filtering, and morphology, etc., relied on OpenCV [52]. Similarly, geometrical operations and displays relied on Visualization Toolkit (VTK) [53].

## 4. Results and Discussion

**Figure 3:**
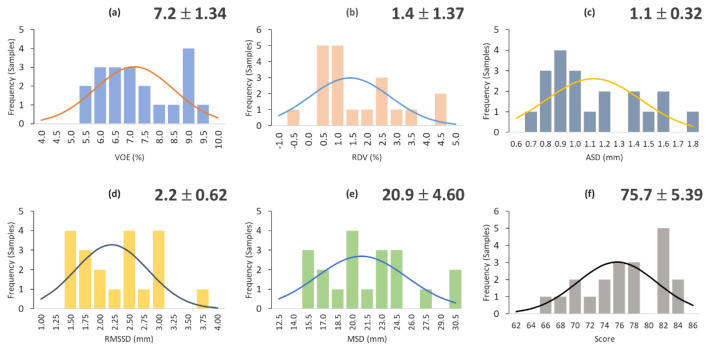
Sample distribution of VOE (**a**), RVD (**b**), ASD (**c**), RMSD (**d**), MSD (**e**) metrics, and overall score (**f**) for all 20 segmented livers from MICCAI SLIVER07.

The distribution of VOE, RVD, ASD, RMSD, MSD metrics, and the overall score for all 20 segmented livers are plotted in Figure 3. The average overall score was 75.69 ± 5.388. The lowest and highest scores were 65.3 and 83.1, respectively. Out of 20 instances, 11 instances gave scores higher than the average.

Subsequently, the evaluation metrics, obtained by the proposed method, for 20 MICCAI images, as well as their processing times, were also compared against those obtained by state-of-the-art methods. The comparisons are listed in Table 2.

It is evident that Liao’s method outperformed the rest and ranked first on VOE, RVD and ASD. Their technique took 4.7 min to segment a liver on average. Meanwhile, Zheng’s work performed best on RMSD and MSD, but worst on RVD and VOE. These findings suggested that the former performed well on volume overlapping, while the latter did so on point-to-surface distance. In terms of processing time, the proposed method was faster than Liao’s and Chen’s, and second only to Wu’s works. However, it gave better VOE and RVD. The improved processing time in our method was primarily due to efficient and intuitive feature extraction. In addition, another key advantage of the proposed method was the lowest standard deviation, which implies the most consistent performance. Its average score was also higher than the reported user (manual) score. This indicates that the proposed method was highly reproducible and could be applied in clinical practice.

**Figure 4:**
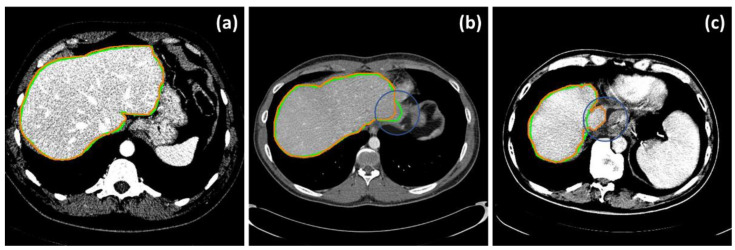
Selected examples of accurate (**a**) and erroneous segmentation, due to an undefined boundary (**b**) and inclusion of the IVC (**c**). Green and orange contours are those of the ground truths and of the livers segmented by the proposed method, respectively.

In addition to the numerical assessments, Figure 4 demonstrated selected instances of highly accurate (Figure 4a) and inaccurate results due, for example, to a low contrast boundary (Figure 4b), and mistakenly including the inferior vena cava (IVC) (Figure 4c), respectively.

**Figure 5:**
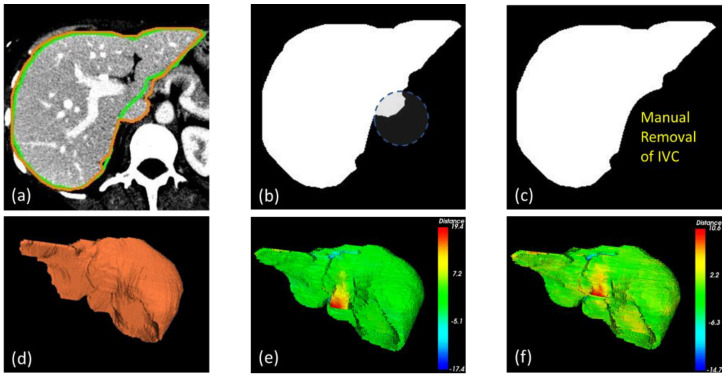
Example of manual IVC removal. Overlaid on an original image (**a**) are the segmented (green) and ground truth (orange) contours. The segmented liver before and after IVC (seen as BN) removal are shown in (**b**,**c**). Compared with the ground truth liver surface (**d**), the one with the IVC removed (**e**) exhibited lower errors [–14.7, 10.6] than did that without (**f**) [–17.4, 19.4].

It should be noted that, without statistical priors on anatomically plausible shapes, it remained challenging to faithfully extract a liver from CT. In addition to vague boundary definition, the mistaken inclusion of the IVC also played a vital part in these errors. By definition [12], vasculature should be included only if it was completely surrounded by the liver. Unfortunately, this was not always the case in a typical dataset, especially for the IVC. In some instances, it was correctly discarded by BN detection, whereas in others, the IVC was gradually merged with the liver and was hence included by interslice consistency constraint. Meticulously fine-tuning the parameters to balance this tradeoff, so as to have the IVC completely removed, is possible but impractical for typical settings. Besides, the inclusion of the IVC merely lessened the performance metrics but did not undermine the quality of the overall shape in many applications. That being said, if a complete separation between a liver and its vasculature is needed, particularly in functional resection and surgical simulation, the IVC can be manually removed on a few 2D slices, before interslice consistency assertion, provided that it was detected as a BN. Figure 5 and Table 3 demonstrate visual and numerical results, before and after manual IVC removal, from a selected example. It was evident that the segmentation was significantly improved after trivial user intervention.

**Figure 6:**
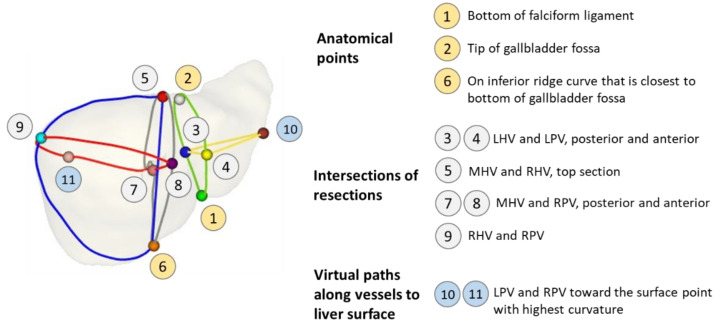
Landmark annotations. LHV, MHV, and RHV refer to the resection paths along the left, middle, and right hepatic veins, respectively; LPV and RPV refer to those along the left and right portal veins, respectively.

In addition, to elucidate the merits of the proposed cross-subject correspondence of liver resection, a pair of extracted liver surfaces were selected and denoted as source (*S*_1_) and target (*S*_2_). After segmentation, these surfaces were first preprocessed by the MeshFix tool [54], to remove degenerated and self-intersecting triangles, to fill holes, and to smooth their vertices. Without a loss of generalization ability, each processed mesh was resampling to contain 12k vertices (to reduce mesh elements to a manageable size, while maintaining visual appearance). Subsequently, functional resection was performed on each liver [55], resulting in eleven landmarks. A description of these landmarks is summarized in Figure 6.

Out of these landmarks, three anatomical points (1, 2, and 6) were explicitly detected on the gallbladder fossa and falciform ligament [55], while the other six points (3–5 and 7–9) were intersections of resection paths; the last two points (10, 11) lay on the virtual paths along the left and right portal veins (PV) passing through the liver surface, where it exhibited the highest curvature.

**Figure 7:**
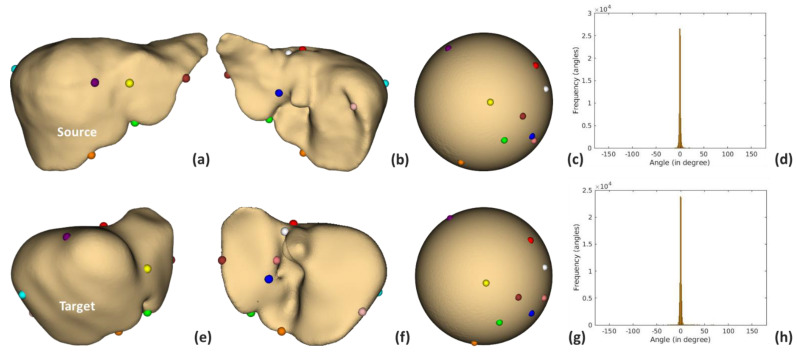
The conformal map with landmark constraints. The first and second rows present the source and target livers in anterior (**a**,**e**) and posterior (**b**,**f**) views. On the spheres in (**c**,**g**) are shown their landmarks, aligned by constrained mapping. The distortions between liver and spherical mapping of source and target livers are plotted in (**d**,**h**), respectively.

**Figure 8:**
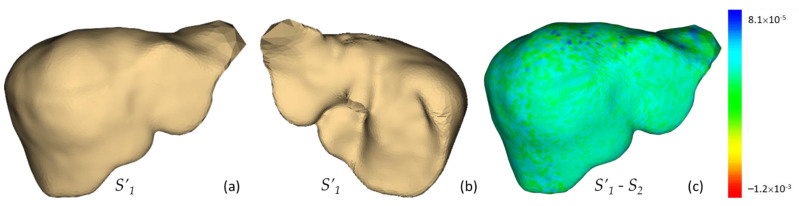
The registration (*S’*_1_) of source (*S*_1_) to target (*S*_2_) in anterior view (**a**), posterior view (**b**), and the overlaid error distance (**c**). The errors of this example were between –1.2 × 10^−3^ to 8.1 × 10^−5^.

Example localization of these landmarks and the respective mapping are illustrated in Figure 7. It is revealed by the distortion plots with a slight deviation from 0 degrees that, by using the FLASH algorithm, stabilized and bijective conformal mapping was obtained. The corresponding source to target registrations are shown in Figure 8.

**Figure 9:**
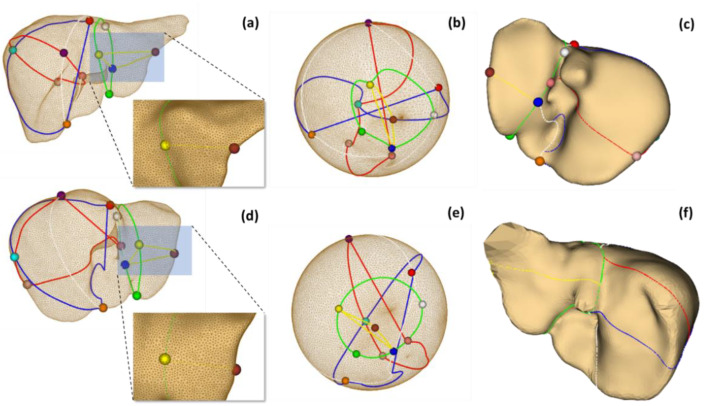
The resection of source (**a**) and target livers (**d**), based on hepatic vascular network, their respective conformal mapping (**b**,**e**), and the projection of target resection (**c**) onto the registered source liver (**f**), by using symmetric correspondence.

To evaluate the correspondence between source and target resections, the resection on the target liver $\left({ℒ}_{2}\right)$, based on its interior vasculature [55] was projected onto the registered source liver, $\left(S_{1}^{’}\right)$, denoted as $\left({ℒ}_{1}^{’}\right)$. Subsequently, the Hausdorff distance between this projected resection and that actually performed on the source $\left({ℒ}_{1}\right)$ was computed by using MeshLab, developed by the Visual Computing Lab of the ISTI-CNR based on the VCG library [56]. Figure 9 depicts the resection on source and target livers [55], their conformal parameterizations, and the projected resection by symmetric correspondences.

Table 4 lists the maximum, minimum, mean and root mean square errors of the Hausdorff distances between the correct (determined from its actual vasculature) and projected resection on the source liver.

It is evident from Table 4 that the projections of LHV and LPV exhibited the least error, which implies they are the most consistent between the projected and correct hepatectomy. In which case, when provided with resection paths on the target liver, that one source could be sufficiently determined by conformal correspondence, without the extraction of the entire hepatic vascular network. On the contrary, those on the right hemi-liver exhibited larger errors. This was due to a greater variation of the corresponding segments (e.g., segments IV and VIII) [55].

It is thus worth emphasizing that the cost function given in Equation (13) is aimed at balancing between minimized distortion and landmark alignment. This implies that exact alignment was not necessarily guaranteed. In fact, in the original FLASH implementation, six sulci, each of which consisted of multiple vertices in a cerebral cortex, were imposed to evenly distribute errors. However, since the liver is much morphologically diverse, localized alignment is suggested. To demonstrate this proposition, five anatomical landmarks relevant to LHV and LPV (i.e., 1, 2, 3, 4, and 10) were imposed as constraints. The corresponding errors between the correct and projected resection are shown in Table 5.

**Figure 10:**
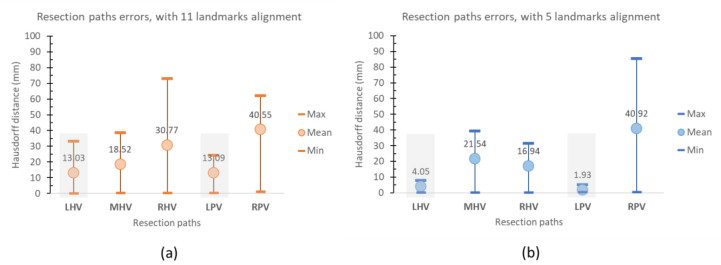
The chart comparing error distances between projected and correct resection paths with 11 (**a**) and 5 (**b**) landmark constraints.

Figure 10 compares these min, max, and mean resection errors between imposing 11 and 5 landmark constraints. It is evident that, by localizing the constraints, the landmarks and hence the relevant resection, i.e., LHV and LPV, were more consistent. However, it could lead to greater errors in other segments, e.g., MHV and RPV paths. Therefore, care must be observed when selecting anatomical landmarks, as they play a vital role in resection consistency.

Unlike [55], the reconstructed segments were no longer limited to planar separation and were hence much more flexible to the actual morphology of an underlying shape. To lessen estimation errors, a statistical atlas of the livers may be built over a training set, and then its mean shape (instead of an arbitrary instance) may be used as a reference [57], while regulating Equation (13) by plausible variation, only found in this set. Its detailed analyses and discussion, however, fall out of the current scope and are left for future investigation.

## 5. Conclusions

Segmenting a liver from a 3D CT image has remained a challenging area of research. This is mainly due to the inherent characteristics of this imaging modality that impede it from rendering clear separation between the liver and connective abdominal parts. In addition, the inhomogeneity of interior voxels has often contributed to erroneous liver extraction.

According to the recent survey, model-based and interactive methods offered by far the most promising results. However, anatomical variations across the subject population and high geometrical resolution call for a prohibitively large, annotated dataset for realistic model construction. On the one hand, given a model that captures all plausible variations, a deformable model approach can be adopted to automatically segment a liver. On the other hand, without such priors, user intervention or empirical knowledge is often required, for example, to initialize seed points, estimate rough contours, or make small adjustments to segmented results.

This research proposed a semi-automatic method for segmenting a liver from CT images. Its main contribution was to balance between robustness, accuracy, processing time, while keeping user interaction minimal. The presented method was extended from a previous attempt [22]. More specifically, the probabilistic model of pixel statistics was first built from a user-defined seed point and then enhanced by multi-slice RL. Subsequently, the liver was extracted using the GC algorithm. During graph optimization, a novel local texture compatibility function called anisotropic measure was integrated into boundary energy to enhance its robustness. Finally, BN detection and contour constraints [22] were employed to resolve the remaining under- and over-segmentation. The experimental results, both visual and numerical, reported herein demonstrated that the proposed method not only gave comparable segmentation accuracy to state-of-the-art methods, but was also faster and more consistent than the majority in its class. It is also worth emphasizing that this favorable performance relied neither on any statistical model nor specific anatomical priors of the liver. It can thus be readily applied to other similar abdominal organs, such as the spleen.

The proposed virtual resection scheme could be integrated with other computerized therapeutic interventions. For example, percutaneous radiofrequency ablation (RFA) is another prospective area of application. This non-invasive treatment first locates the tumor and then precisely destroys it by heat through a needle. To this end, Egger et al. [58] constructed a 3D spherical graph at a seed point. Subsequently, max-flow min-cuts was used to segment the ablation zone from the background. An automatic approach was taken by Wu et al. [59]. With their method, fuzzy c-mean clustering and cyclic morphology were employed to extract and then refine the ablation zone. Having this zone extracted (or during the procedure), the proposed scheme could be applied to determine the enclosing liver and functional segments, to gain a comprehensive view of the overall treatment.

Thus far, there remain limitations with the proposed method worth investigating in the future. One of them is complete IVC removal, which is vital for preoperative planning and computerized surgical simulation. This can be done by segmenting the liver and its vasculature simultaneously. Furthermore, errors may be propagated during post-processing. A novel user experience (UX) and interface (UI) design could help make manual editing much more intuitive and efficient. Lastly, the proposed method did require a seed point from a user. To fully automate the segmentation, the seed point may be chosen, such that the resultant probabilistic map virtually covers an estimated VOI.

After that stage, we demonstrated that the parametric functional segments of a liver could be reconstructed by a conformal mapping of its extracted surface on a genus-0 domain. Its potential application was in estimating functional resection on one liver when provided with that on another, and with a few landmarks specified on both shapes. Such symmetric correspondence would be useful, for instance, in a preoperative simulation of a living donor liver transplant. With the proposed scheme, a partial hepatectomy between individuals could be rapidly determined, based on explicit biomarkers and the conformal properties of relevant shapes. Since the resection was no longer limited by planar intersections, as imposed in previous works, they were much more adaptive to liver morphology. In the future, we plan to build an atlas of plausible functional segments, for much more reliable and robust correspondences against pathologies.

## Figures and Tables

**Table 1:** Summary of the recent liver segmentation methods in the literature.

Reference	Method	Data	Results	Auto
Chen et al., 2009 [16]	Region growing (RG)	Private	Visual assessment	No
Kumar et al., 2011 [17]	Largest connected region and RG	Private	Average absolute volume error: 1.52%, Mean spatial overlap ± standard deviation: 0.98 ± 0.01	Yes
Chen et al., 2012 [21]	AAM, Live wire, Graph cut (GC)	MICCAI (Labeled)	VOE: 6.5 ± 1.8%	Yes
Lu et al., 2014 [13]	Quasi-Monte Carlo (QMC)	2D images	Visual assessment	No
Yang et al., 2014 [25]	Level-sets	MICCAI (Unlabeled) Private	Score: 78.9 SI = 97.6%	No
Peng et al., 2015 [19]	Multiple region appearances and GC	MICCAI, Private	Score: 83.4 and 83.4 DSC: 97.5% ± 0.4%	No
Huang et al., 2016 [15]	SBLDA	3Dircadb1	VOE: 7.84 ± 2.95%	Yes
Liao et al., 2016 [5]	GC and bottleneck detection	MICCAI, XHCSU14	VOE: 5.8 ± 3.3% VOE: 5.8 ± 0.7%	No
Wu et al., 2016 [20]	Simple linear iterative clustering (SLIC) and super voxel-based GC	MICCAI	Score: 75.2 and 71.4	Yes
Mohamed et al., 2017 [11]	Largest connected component (LCC) and Bayesian model	Private	DSC: 95.5%	Yes
Liao et al., 2017 [18]	GC and border marching	MICCAI, XHCSU14	VOE: 5.8 ± 3.2% VOE: 5.4 ± 0.7%	Yes
Lu et al., 2017 [23]	3D CNN and GC	MICCAI, 3Dircadb1	Score 77.8 VOE: 9.36 ± 3.34%	No
Zheng et al., 2017 [24]	Random walk	MICCAI (Labeled)	VOE: 7.8%, Score: 76	Yes
Chartrand et al., 2017 [26]	Deformation model using Laplacian mesh optimization scheme	MICCAI, Private	VOE: 5.14 ± 1.02% VOE: 5.1 ± 0.6% VOE: 7.62 ± 1.35%	No
Le et al., 2021 [22]	MND, GC and anatomical constraints	MICCAI	VOE: 7.8 ± 1.5% VOE: 8.0 ± 1.1%	No

**Table 2:** Benchmarking on five standard metrics and processing time, among different methods.

Metrics	VOE	RVD	ASD	RMSD	MSD	Running Time
Methods	[%]	[%]	[mm]	[mm]	[mm]	[min]
Liao et al. [18]	5.8 ± 3.2	−0.1 ± 4.1	1.0 ± 0.5	2.0 ± 1.2	21.2 ± 9.3	4.7
Chen et al. [21]	6.5 ± 1.8	−2.1 ± 2.3	1.0 ± 0.4	1.8 ± 1.0	20.5 ± 9.3	6.0
Wu et al. [20]	7.5 ± *	4.2 ± *	1.0 ± *	1.9 ± *	18.5 ± *	0.4
Zheng et al. [24]	7.8 ± *	5.1 ± *	1.1 ± *	1.4 ± *	11.1 ± *	*
Proposed method	7.2 ± 1.3	1.4 ± 1.4	1.1 ± 0.3	2.2 ± 0.6	20.9 ± 4.6	1.4

Note: Fields denoted with * are those not reported in the corresponding works.

**Table 3:** The metrics and overall scores of the above example, before and after removing IVC.

Metrics	VOE [%]	RVD [%]	ASD [mm]	RMSD [mm]	MSD [mm]	Overall Score
Before IVC removal	6.9	3.2	0.9	1.8	19.4	76.7
After IVC removal	6.4	2.7	0.8	1.4	14.7	80.6

**Table 4:** The Hausdorff distance between the correct and projected hepatectomy with 11 landmarks.

Resection Paths	Max [mm]	Min [mm]	Mean [mm]	RMS [mm]
LHV	33.12	0.15	13.03	15.50
MHV	38.53	0.45	18.52	21.54
RHV	72.77	0.36	30.77	36.32
LPV	24.10	0.23	13.09	14.32
RPV	62.12	1.06	40.55	43.56
Average	46.130	0.452	23.192	26.249

**Table 5:** The Hausdorff distance between the correct and projected hepatectomy with 5 landmarks.

Resection Paths	Max [mm]	Min [mm]	Mean [mm]	RMS [mm]
LHV	7.85	0.12	4.05	4.44
MHV	39.18	0.16	21.54	23.81
RHV	31.27	0.31	16.94	19.48
LPV	5.13	0.34	1.94	2.09
RPV	85.42	0.39	40.92	48.27
Average	33.707	0.266	17.074	19.618

## Data Availability

Volumetric CT images of livers employed in this study were obtained from MICCAI SLIVER07 dataset. It was available at http://www.sliver07.org/, accessed on 27 August 2018.
